# Antibiotic Prophylaxis in Prostate Biopsies: Contemporary Practice Patterns in Germany

**DOI:** 10.3389/fsurg.2018.00002

**Published:** 2018-01-24

**Authors:** Katharina Boehm, Fabian P. Siegel, Laila Schneidewind, Jennifer Kranz, Philipp Spachmann, Tanja Frank, Nina Huck, Florian Imkamp, Alexandre Pelzer

**Affiliations:** ^1^Universitätsmedizin Mainz, Klinik und Poliklinik für Urologie und Kinderurologie, Mainz, Germany; ^2^Universitätsmedizin Mannheim, Klinik für Urologie, Mannheim, Germany; ^3^Universitätsmedizin Greifswald, Klinik für Innere Medizin C, Hämatologie/Onkologie, Greifswald, Germany; ^4^St.-Antonius Hospital, Klinik für Urologie und Kinderurologie, Akademisches Lehrkrankenhaus der RWTH Aachen, Eschweiler, Germany; ^5^Caritas Krankenhaus St. Josef, Klinik für Urologie, Universität Regensburg, Regensburg, Germany; ^6^Klinikum Rosenheim, Klinik für Urologie und Kinderurologie, Rosenheim, Germany; ^7^Medizinische Hochschule Hannover, Klinik für Urologie und urologische Onkologie, Hannover, Germany; ^8^Department of Urology, Hospital Wels-Grieskirchen, Wels, Austria

**Keywords:** prostate biopsy, healthcare research, antibiotic prophylaxis, antibiotic stewardship, transrectal biopsy, perineal biopsy

## Abstract

**Purpose:**

Prostate biopsy (pbx) is the most common outpatient procedure in urology. Complications are urinary tract infections, including hospitalization and sepsis. Recommendations on antibiotic prophylaxis (apx) are scarce, and healthcare data are not available. The study addressed the following endpoints: the duration and spectrum of antimicrobial prophylaxis in transrectal and transperineal pbx in the hospital and the practice setting.

**Methods:**

A questionnaire compiled data about age, gender, board certification, and place of work. Information about the frequency of pbx, duration and type of apx, usage of disinfecting lubricant, and urine or rectal swab cultures was collected. The study refers to German urologists.

**Results:**

Overall 478 urologists answered the questionnaire. 15.5% (74) of respondents were residents. 50.8% (243) of urologists work in a practice; the rest in a hospital. Only 4.8% do not perform pbx. Transrectal pbx are performed a median of two times a week. The majority (446, 98%) prescribe an apx, mostly fluoroquinolones (407, 89.5%). In total, 10.1% (46) of the participants use a single-shot-apx. apx has a median duration of 4 days. One-third uses a disinfecting lubricant. Urine and rectal swab cultures are analyzed by 45.5% (207) and 24.4% (111), respectively.

**Conclusion:**

Most urologists prescribe an extended apx for both transrectal and transperineal pbx. Perineal pbx is still a deviation from everyday practice and not an established alternative to transrectal pbx. Urologists are aware of the increasing fluoroquinolone-resistance and are adapting with rectal swab and urine cultures. Further studies need to evaluate alternatives to 5-day apx and results should be addressed in our guidelines. This is of importance in light of the increasing resistance rates and fluoroquinolone side effects.

## Introduction

Prostate biopsy (pbx) is the most frequently performed outpatient procedure in urology. At an incidence of about 70,000 per year (1) and a rough estimate of about twice the number of prostate biopsies a year, about 140,000 biopsies are performed every year in Germany. Prostate biopsies are performed not solely for diagnosing prostate cancer but also for active surveillance strategies.

However, this standard procedure is associated with several risks. Post-interventional complications include urogenital infections in 1.0–17.5% of cases, hematuria in about 65.8%, and a worsening of lower urinary tract symptoms in up to 25% (2). Urogenital infections can lead to hospitalization and sepsis, making pbx cost intensive. In accordance with the current national and international guidelines antimicrobial prophylaxis is recommended, most common are fluoroquinolones (3). There are no definitive recommendations concerning duration or choice of regimen (3). There is evidence that fluoroquinolone resistance is increasing to 10–30% (2), which prompts urologists to search for alternatives. For example, perineal biopsies might be associated with a lower risk of infectious complications compared to transrectal biopsies (4).

Infectious prophylaxis is usually initiated before a pbx. There is currently no evidence concerning the duration and type of antibiotic prophylaxis (apx) (5). It is unclear what kind of apx is performed for transrectal biopsy in German hospitals and practices. In addition, no data are available about perineal biopsies or additional procedures like, e.g., rectal swabs, local antimicrobial cleansing. The aim of this descriptive study was to evaluate the duration and spectrum of antimicrobial prophylaxis in transrectal and transperineal pbx in the hospital and the practice setting.

## Materials and Methods

An online questionnaire was created on www.surveymonkey.de. It was distributed *via* the email lists of the German Association of Urology [Deutsche Gesellschaft für Urologie (DGU)], the Association of German Urologists [Berufsverband der Deutschen Urologen (BDU)], and the German Society of Residents in Urology (GeSRU). A printed version was distributed at five regional meetings.

The questionnaire consisted of 22 questions. Demographic data such as age, gender, the size of practice/hospital, and work experience were collected as multiple choice questions. Details about the performance of prostate biopsies were compiled (apx, duration of prophylaxis, use of local antimicrobial lubricant, urine cultures, and rectal swabs). Questions about the type of antibiotics were kept as free text questions. Questions were specifically adapted for transrectal or perineal pbx.

Statistical analysis was performed using R Version 3.1.0 (www.r-project.org) (6).

Continuous data were analyzed by using Student’s *t*-test, ordinal data by Mann–Whitney–Wilcoxon test, and categorical data by χ^2^-test. Statistical significance was defined as *p* < 0.05. Missing data were marked as NA.

## Results

A total of 478 urologists completed the questionnaire (84.7% males, 13.4% females). Because of an overlap in the email lists, a response rate could not be calculated. 74 (15.5%) of the respondents were residents. 243 (50.8%) urologists work in a practice, and 235 (49.2%) in a hospital. Only 23 (4.5%) of participants did not perform biopsies themselves (Table 1). Transrectal biopsies are performed by 455 (95.2%) of the participants, whereas transperineal biopsies are performed by 75 (1.6%) of the participants.

**Table 1:** Descriptive characteristics of the participants (*n* = 478).

Variables	Overall	Biopsy yes	Biopsy no	*p*-Value
*n* (%)	478	455	23	
Age (years), mean, median (IQR)	47, 48 (39–55)	47, 48 (39–55)	47, 47 (30–64)	0.9
Start of practice (years), mean, median (IQR)	2001, 2002 (1994–2007)	2002, 2002 (1994–2007)	1993, 1992 (1983–2003)	0.045
Gender, *n* (%)				
– Female	64 (13.4%)	60 (13.2%)	4 (17.4%)	
– Male	405 (84.7%)	386 (84.8%)	19 (82.6%)	
– NA	9 (1.9%)	9 (2%)	0 (0%)	
Work place, *n* (%)				0.051
– Practice	243 (50.8%)	237 (52.1%)	6 (26.1%)	
– Hospital of primary or secondary health care	127 (26.6%)	118 (25.9%)	9 (39.1%)	
– Hospital of tertiary health care/university hospital	108 (22.6%)	100 (22%)	8 (34.8%)	
Region, *n* (%)				0.006
– Urban (<20,000 inhabitants)	51 (10.7%)	50 (11%)	1 (4.3%)	
– Rural (20–100,000 inhabitants)	173 (36.2%)	168 (36.9%)	5 (21.7%)	
– Metropolitan (>100,000 inhabitants)	252 (52.7%)	236 (51.9%)	16 (69.6%)	
Educational status, *n* (%)				0.02
– Resident	74 (15.5%)	66 (14.5%)	8 (34.8%)	
– Certified urologist	404 (84.5%)	389 (85.5%)	15 (65.2%)	

*Respondents were stratified by performing prostate biopsy (pbx) vs. not performing pbx. Participants were questioned between June 2015 and March 2016*.

### Transrectal Biopsies

A total of 455 urologists perform transrectal prostate biopsies (Table 2). 237 (52.1%) of them work in hospitals. A median of two biopsies is performed per week (IQR 0.8–3) (Table 2). Urologists in hospitals perform significantly more biopsies than colleagues in a practice (median 2.7 vs. 1.2; *p* < 0.001; Table 2).

**Table 2:** Antibiotic prophylaxis (apx) and supportive care in participants performing transrectal biopsy.

Variables	Overall	Hospital	Practice	*p*-Value
*n* (%)	455	237	218	
No biopsy per week, mean, median (IQR)	7.5, 2 (0.8–3)	14, 2.7 (1.1–5)	1.8, 1.2 (0.8–2)	<0.001
apx, *n* (%)				0.03
– Yes	446 (98%)	211 (96.8%)	235 (99.2)	
– No	3 (0.7%)	1 (0.5%)	2 (0.8)	
– NA	6 (1.3%)	6 (2.8%)	0 (0)	
Duration of apx (days), mean, median (IQR)	3.8, 4 (2–5)	3.5, 3 (2–4)	4.1, 4 (3–5)	<0.001
Type of apx, *n* (%)				0.005
– Fluoroquinolones	407 (89.5%)	187 (85.8%)	220 (92.8%)	
– Others	18 (4%)	8 (3.7%)	10 (4.2%)	
– NA	30 (6.6%)	23 (10.6%)	7 (3%)	
Single shot, *n* (%)				<0.001
– Yes	46 (10.1%)	36 (16.5%)	10 (4.2)	
– No	409 (89.9%)	182 (83.5%)	227 (95.8)	
Antimicrobial lubricant, *n* (%)				<0.001
– Yes	147 (32.3%)	49 (22.5%)	98 (41.4)	
– No	274 (60.2%)	142 (65.1%)	132 (55.7)	
Rectal swab, *n* (%)				<0.001
– Yes	111 (24.4%)	52 (23.9%)	59 (24.9%)	
– No	315 (69.2%)	142 (65.1%)	173 (73%)	
– NA	29 (6.4%)	24 (11%)	5 (2.1%)	
Urine culture, *n* (%)				<0.001
– Yes	207 (45.5%)	101 (46.3%)	106 (44.7%)	
– No	219 (48.1%)	93 (42.7%)	126 (53.2%)	
– NA	29 (6.4%)	24 (11%)	5 (2.1%)	

*Results are stratified according to work place hospital vs. practice*.

The overwhelming majority of 446 (98.0%) respondents perform an apx; only 3 (0.7%) do not (*p* < 0.001). The antibiotic agent of choice is a quinolone (407 [89.5%]). The other urologists use gentamicin, fosfomycin, ampicillin/sulbactam, or did not specify the agent. Urologists working in a practice setting used quinolones even more frequently compared to urologists in hospitals (220 [92.8%] vs. 187 [85.8%], *p* = 0.005). Only 46 (10.1%) urologists administer a single-shot prophylaxis, this was significantly more common in the hospital setting than in the urological practice (16.5 vs. 4.2%; *p* < 0.001). apx is given a median of 4 days (IQR 2–5) (3 days in hospitals vs. 4 days in practices, *p* < 0.001).

In total, 141 [33.1%] urologists used an antimicrobial lubricant. This was significantly more common in practices than in hospitals (41.4 vs. 22.5%, *p* < 0.001). Pre-interventional urine cultures and rectal swabs are collected by 190 (44.6%) and 102 (23.9%), respectively (Table 2).

### Transperineal Biopsies

A total of 75 urologists perform perineal prostate biopsies (Table 3), with 59 (78.7%) of whom work in hospitals. There is no significant difference in any metric between urologists in hospitals or practices. A median of three biopsies is performed per week (IQR 2–5). An apx is performed by 88% of respondents (*n* = 66); the remaining nine participants did not answer this question. apx is given a median of 3 days (IQR 2–4) and consists primarily of fluoroquinolones (*n* = 65 [86.7%]) without significant differences between urologists in hospitals or practices (*n* = 51 [86.4%] vs. 14 [87.5%]; *p* = 0.9). One urologist does not use a fixed antibiotic regimen but instead chooses the antibiotic according to the rectal flora of the patient. Nine participants (12%) give a single-shot prophylaxis. No statistically significant difference could be shown between urologists working in hospitals vs. those working in practices (8 [13.6%] vs. 1 [6.2%], *p* = 0.7). Urine cultures are performed by 35 (46.7%) of the urologists (30 [50.8%] vs. 5 [31.2%], *p* = 0.3).

**Table 3:** Antibiotic prophylaxis (apx) and supportive procedures in participants performing perineal biopsy.

Variables	Overall	Hospital	Practice	*p*-Value
*n* (%)	75	59	16	
No biopsy per week, mean, median (IQR)	4.9, 3 (2–5)	5.4, 3.9 (2–5)	3.3, 3 (1.5–4)	0.2
apx, *n* (%)				0.03
– Yes	66 (88%)	52 (88.1%)	14 (87.5%)	
– No	0 (0%)	0 (0%)	0 (0%)	
– NA	9 (12%)	7 (11.9%)	2 (12.5%)	
Duration of apx (days), mean, median (IQR)	3.5, 3 (2–4)	3.5, 3 (2–4)	3.5, 3 (3–5)	0.5
Type of apx, *n* (%)				0.9
– Fluoroquinolones	65 (86.7%)	51 (86.4%)	14 (87.5%)	
– Others	1 (1.3%)	1 (1.7%)	0 (0%)	
– NA	9 (12%)	7 (11.9%)	2 (12.5%)	
Single shot, *n* (%)				0.7
– Yes	9 (12%)	8 (13.6%)	1 (6.2%)	
– No	66 (88%)	51 (86.4%)	15 (93.8%)	
Urine culture, *n* (%)				0.3
– Yes	35 (46.7%)	30 (50.8%)	5 (31.2%)	
– No	31 (41.3%)	22 (37.3%)	9 (56.2%)	
– NA	9 (12%)	7 (11.9%)	2 (12.5%)	

*Results are stratified according to work place hospital vs. practice*.

## Discussion

Our hypothesis states that currently no evidence concerning the duration and type of antimicrobial prophylaxis exists.

Our key findings are German urologists participating in the study seem to prefer an apx of several days (89.9%) to a single-shot (10.1%) prophylaxis. However, many urologists are aware of the increased risk of antibiotic resistance and perform additional tests such as urine cultures and rectal swabs (44.6 and 23.9%, respectively). A significant proportion (33.1%) is using an antimicrobial lubricant.

Povidone-iodine rectal cleansing in combination with targeted antimicrobial prophylaxis using rectal swab cultures can reduce infectious complications (7). Use of an antimicrobial lubricant has been shown to reduce bacterial count in rectal swab cultures (8). A meta-analysis including 1,373 patients has demonstrated a lower rate of infectious complications after a povidone-iodine rectal cleansing and is therefore recommended by the EAU guideline (5).

The 2015 version of the EAU guidelines on urological infections recommended a single-day or single-shot prophylaxis in low-risk patients (9). The current guideline does not give a definitive suggestion on this (3), as the influence of duration and the class of antibiotic on infectious complications is unclear. A 2011 Cochrane analysis could not show a significant difference in serious complications between a single-dose and a multiple-dose prophylaxis (10). This suggests restraint in prescribing a multi-dose-prophylaxis in low-risk patients. In high-risk patients, however, the development is different: some advocate combining an augmented prophylaxis with >2 antibiotics with povidone-iodine bowel preparation in high-risk regions (11). Others combine ciprofloxacin with amoxicillin/clavulanate (12) or administer meropenem (13) for antimicrobial prophylaxis in high-risk patients.

The risk categories are not strictly defined and are mostly based on the probability of resistance to fluoroquinolones: The usage of fluoroquinolones for prophylaxis is supported by the EAU guideline (3). However, fluoroquinolone-resistant bacteria have been found in 10–30% of patients in pre-interventional rectal swab cultures (2). It has been shown that ciprofloxacin resistance in rectal swab cultures, as well as a history of taking ciprofloxacin during the three preceding months before biopsy, increases the risk of infectious complications (14). The Global Prevalence of Infections in Urology study has demonstrated an increase in fluoroquinolone-resistant bacteria after having traveled to countries with a high incidence of fluoroquinolone-resistant bacteria and a history of fluoroquinolone intake during the preceding 6 months (15, 16). Therefore, performing a rectal swab might be useful at least in high-risk patients. Generally, resistance rates differ from area to area; it can be prudent to evaluate local resistance rates by rectal swab culture. While the incidence of sepsis can be reduced by a targeted prophylaxis (17), the quality of evidence is currently up for debate (18). In our cohort, rectal swabs are performed by a quarter of respondents.

Other risk factors for infectious complications are comorbidities, age, diabetes, and prostate enlargement (2, 19). The hospitalization rate is reported to be less than 5% (19). Nonetheless, the incidence of sepsis has increased from 0.52% (2002–2009) to 2.15% (2010–2011) (20). This might be linked to higher antibiotic resistance rates, which have been increasing continuously during the past years (21). Taken together, it seems prudent to refine the choice and length of apx prior to pbx.

To further multiply the disadvantages of the current apx regimen the Food and Drug Administration issued a black box warning for all fluoroquinolones in July 2016. The rationale behind this was the reassessment of serious adverse reactions such as tendinitis including tendon rupture, peripheral neuropathy, central nervous system effects, and exacerbation of myasthenia gravis (22). In consequence, fluoroquinolones should only be used when there are no other treatment options available. As a result, the efficacy of other antibiotics in prophylaxis should be evaluated.

According to the current guidelines on urological infections, the length of apx should be reduced to a minimum (3). A prolonged prophylaxis is generally recommended in patients at high risk for infectious complications. The median of fluoroquinolone administration in this study was 4 days. This extended regimen might be cost intensive, related to side effects for the patient, and lead to higher resistance rates, without adding a substantial benefit. Therefore, duration of apx should be addressed in the guidelines.

Although theoretically sound, the benefit of a urine culture before pbx has not yet been shown (23). Considering the possible exacerbation of a dormant or subclinical bacterial prostatitis, it seems prudent to screen for urinary tract infections prior to pbx. This is done by 45.5% of the participants.

There is an ongoing discussion whether the perineal approach might help to overcome the unsolved issues regarding infectious complications and bacterial resistance. It remains unclear whether a transperineal pbx is a solution to this quandary. At least German urologists do not seem to be convinced, as no urologists abandoned prolonged apx. The rate of single-shot-administration of antibiotics is similar to transrectal biopsy (10.1 vs. 12.0%). The choice of antibiotics is even less diverse than in transrectal biopsy. Some authors report lower rates of severe infectious complications for the transperineal approach (4, 24, 25). In contrast, a 2012 meta-analysis has not demonstrated any difference in complications between transrectal and transperineal biopsies (26) and a 2013 report has shown infection rates similar to transrectal pbx (27). A major disadvantage of perineal pbx is the need for general anesthesia whereas transrectal pbx is usually performed with local anesthesia or even without any pain medication.

Our study has several strengths. First, to the best of our knowledge, it is the first study evaluating everyday practice in a large cohort of German urologists. Second, our study design has allowed to answer questions relevant to healthcare research and shows an uncertainty among wide parts of German urologists regarding specifically the lengths of apx. One major finding is that perineal pbx is still a deviation from everyday practice and far from being an established alternative.

The main limitation of our study is a selection bias. Although our questionnaire was sent to an extensive email list of DGU, BDU, and GeSRU, responding doctors are probably more interested in apx and antibiotic stewardship than the common urologist. Also, the questionnaire only asked about a person’s personal approach. Multiple answers from one institution are therefore possible. The email lists of the DGU, BDU, and GeSRU show significant overlap. Therefore, one of the major limitations of our study was that we were unable to calculate the return rate, due to the study design. In 2016, there were about 5,900 board-certified urologists in Germany (28). Assuming an equal number of urological residents, there are about 12,000 urologic physicians in Germany. The return rate could then be estimated as 4%.

## Conclusion

A majority of urologists prescribe an extended apx for both transrectal and transperineal pbx. Single-shot prophylaxis is slightly more prevalent in hospitals than in urological practices, whereas the use of antimicrobial lubricants is more common in the practice setting. One major finding is that perineal pbx is still a deviation from everyday practice and far from being an established alternative to transrectal biopsy. Urologists are aware of the increasing fluoroquinolone-resistance and are adapting with a rectal swab and urine cultures.

Further studies need to evaluate alternatives to 5-day-antibiotic “therapies” disguised as a prophylaxis and results should be addressed in our guidelines. This is of utmost importance in light of the increasing resistance rates and fluoroquinolone side effects.

## Ethics Statement

Research involving human participants and/or animals: the study did not include data on human participants or animals. Informed consent: all data were collected *via* an email questionnaire on a voluntary base, ethics committee approval is not required as per the local legislation.

## Author Contributions

Conception and design of the work: KB, LS, JK, and FI. Acquisition of data: KB, NH, TF, and FS. Analysis of data: KB and FS. Interpretation of data for the work: KB, FS, and AP. PS contributed in Protocol/project development and manuscript writing/editing. All authors were drafting the work or revising it critically for important intellectual content; gave final approval of the version to be published; gave their agreement to be accountable for all aspects of the work in ensuring that questions related to the accuracy or integrity of any part of the work are appropriately investigated and resolved.

## Conflict of Interest Statement

The authors declare that the research was conducted in the absence of any commercial or financial relationships that could be construed as a potential conflict of interest. The reviewer PM declared a past co-authorship with one of the authors KB to the handling editor.
