# Supplementary material for: Antibiotic Prophylaxis in Prostate Biopsies: Contemporary Practice Patterns in Germany
Source: Front Surg. 2018 Jan 24;5:2. doi: 10.3389/fsurg.2018.00002 (PMC5787537; doi:10.3389/fsurg.2018.00002)
Supplement: Supplementary file 1 [file data_sheet_1.docx]

**Questionnaire**

1. **Demographical Questions**
   1. **Age**
   2. **Gender:** ⧠ female, ⧠ male
   3. ⧠ practice
      1. ⧠ practice with one urologist
      2. ⧠ group practice
      3. ⧠ associated practices

⧠ clinic

- - 1. ⧠ Primary-/secondary care
    2. ⧠ Tertiary care
    3. ⧠ university clinic
  1. **For how many years have you been working?** ____________
  2. **Is your catchment area?**
     1. ⧠ rural
     2. ⧠ urban
  3. **In which province do you work?**
     1. Bavaria
     2. Baden-Württemberg
     3. Berlin
     4. Brandenburg
     5. Bremen
     6. Hamburg
     7. Hesse
     8. Mecklenburg-West Pomerania
     9. Lower Saxony
     10. North Rhine-Westphalia
     11. Rhineland-Palatinate
     12. Saarland
     13. Saxony
     14. Saxony-Anhalt
     15. Schleswig-Holstein
     16. Thuringia

1. **Do you personally perfom transrectal prostate biopsies?**

⧠ Yes, ⧠ no

if yes, how often?

- - 1. ⧠ daily
    2. ⧠ weekly
    3. ⧠ monthly
    4. ⧠ ___/day
    5. ⧠ ___/week
    6. ⧠ ___/month

1. **Do you administer antimicrobial prophylaxis in transrectal biopsies?**

⧠ yes, ⧠ no

**If yes, which antibiotic?**

- - 1. ⧠ *[antibiotic] [dosage] [frequency]*

starting ___ days before biopsy to ___ days after biopsy

- - - 1. choice box *antibiotic*:
         1. Ciprofloxacin
         2. Levofloxacin
         3. Amoxicillin/Clavulanic acid
         4. Ampicillin/Sulbactam
         5. Cotrimoxazole
         6. Cefpodoxime
         7. Nitrofurantoin
         8. Fosfomycin
         9. Penicillin
         10. Imipenem
         11. Gentamicin
         12. Metronidazole
         13. other: __________
      2. choice box *Dosage*: includes common dosages for the selected drug. e.g.
         1. 500mg
         2. 250mg
         3. other: _______
      3. choice box *frequency*:
         1. 1x per day
         2. 2x per day
         3. 3x per day
         4. other: ______
    1. ⧠ Singleshot on the day of the biopsy
       1. choice box *antibiotic* (see above)
       2. Dosage: ______ mg

1. **Under what circumstances do you deviate from the standard?**
   1. **Allergies/intolerances:** ⧠ yes, ⧠ no
      1. if yes, how do you do it? ______________
   2. **Endocarditis prophylaxis**: ⧠ yes, ⧠ no
      1. if yes, how do you do it? ______________
   3. **History of sepsis/infection after biopsy:** ⧠ yes, ⧠ no
      1. if yes, how do you do it? ______________
   4. **Risk factors for resistance to fluoroquinolones,** e.g.:_________
   5. **other:** ___________________
2. **Do you use an antimicrobial lubricant?** ⧠ yes, ⧠ no
3. **Do you collect urine culture before a biopsy?**

⧠ yes, ⧠ no

**If yes?**

- - 1. ⧠ Always
    2. ⧠ if risk factors are present. e.g. _______________

1. **Do you collect rectal swabs before a transrectal biopsy?**

⧠ yes, ⧠ no

**If yes?**

- - 1. ⧠ Always
    2. ⧠ if risk factors are present. e.g._______________

1. **Do you personally perform perineal prostate biopsies?**

*Answers identical to question 2*

1. **Do you administer antimicrobial prophylaxis in perineal biopsies**

*Answers identical to question 3*

1. **Under what circumstances do you deviate from the standard?**

*Answers identical to question 4*
